# A Comprehensive Review of the Antitumor Properties and Mechanistic Insights of Duocarmycin Analogs

**DOI:** 10.3390/cancers16193293

**Published:** 2024-09-27

**Authors:** Ann Morcos, Yeonkyu Jung, Joab Galvan Bustillos, Ryan N. Fuller, David Caba Molina, Antonella Bertucci, Kristopher E. Boyle, Marcelo E. Vazquez, Nathan R. Wall

**Affiliations:** 1 Department of Radiation Medicine, James M. Slater, MD Proton Treatment & Research Center, Loma Linda University Health, Loma Linda, CA 92350, USA; 2 Division of Biochemistry, Department of Basic Science, Loma Linda University School of Medicine, Loma Linda, CA 92350, USA; 3 Division of Surgical Oncology, Department of Surgery, Loma Linda University Health, Loma Linda, CA 92350, USA; dcabamolina@llu.edu; 4 Nuclear Response & Analysis, Canadian Nuclear Laboratories, Chalk River, ON K0J 1J0, Canada; 5 Loma Linda University School of Pharmacy, Loma Linda, CA 92399, USA; 6 Radiobiology & Health, Canadian Nuclear Laboratories, Chalk River, ON K0J 1J0, Canada

**Keywords:** duocarmycin, seco-DSA, potency, resistance, cellular mechanisms

## Abstract

**Simple Summary:**

The duocarmycin family consists of highly potent cytotoxic agents originally derived from the bacterium Streptomyces. This review explores their unique chemical structures, which include a DNA-binding unit, a subunit-linking amide for positioning within the DNA helix, and an alkylating unit that targets adenine bases. Duocarmycins disrupt DNA replication and transcription by forming covalent bonds with DNA. Recent advances, such as the development of antibody–drug conjugates (ADCs), have enhanced the potential for targeting cancer cells more effectively. Although preclinical studies show promise, further research is needed to understand the toxicology of duocarmycins before they can be widely applied in cancer treatment. This review summarizes key findings and suggests next steps for improving duocarmycin-based therapies.

**Abstract:**

The duocarmycin family is a group of potent cytotoxic agents originally isolated from the bacterium Streptomyces. This discovery has spurred significant interest due to duocarmycins’ unique chemical structures and powerful mechanism of action. This review comprehensively details the history of the duocarmycin family, the current understanding of their therapeutic potential, and the major clinical trials that have been conducted. Chemically, the duocarmycin family is characterized by a DNA-binding unit that confers specificity, a subunit-linking amide that positions the molecule within the DNA helix, and an alkylating unit that interacts with the DNA. This configuration allows them to bind selectively to the minor groove of DNA and alkylate adenine bases, a notable deviation from the more common guanine targeting performed by other alkylating agents. Duocarmycin’s mechanism of action involves the formation of covalent adducts with DNA, leading to the disruption of the DNA architecture and subsequent inhibition of replication and transcription. Recent advancements in drug delivery systems, such as antibody–drug conjugates (ADCs), have further elevated the therapeutic prospects of duocarmycin analogs by providing a promising mechanism for enhancing intracellular concentrations and selective tumor delivery. Preclinical studies have highlighted the efficacy of duocarmycin derivatives in various in vitro models, providing a strong foundation for translational research. However, further biological research is required to fully understand the toxicology of duocarmycin family members before it can be clinically relevant. The major focus of this review is to cache the major biologically relevant findings of different duocarmycin analogs as well as their biological shortcomings to propose next steps in the field of cancer therapy with these potent therapeutics.

## 1. Introduction

The relentless pursuit of effective cancer therapies has led to the exploration and utilization of various classes of cytotoxic agents, among which the duocarmycin family has emerged as a potent group [1]. Originating from natural sources [2,3], duocarmycins are distinguished by their ability to covalently bind DNA [4] and induce significant cytotoxic effects even at picomolar concentrations [5]. This unique capability positions them as invaluable tools in the fight against cancer, particularly due to their mechanism of action, which involves alkylation of the DNA [3], leading to the disruption of the cancer cell’s replication process.

Alkylating agents represent a cornerstone in the arsenal of cancer chemotherapy due to their ability to add alkyl groups to many electronegative DNA moieties under conditions present in cells [6]. By forming covalent bonds with DNA bases, alkylating agents prevent cancer cells from dividing and proliferating uncontrollably, leading to cell death. These agents are used to treat a variety of cancers, making them central to chemotherapeutic strategies. Alkylating agents’ importance lies in their ability to interfere with DNA-mediated cellular processes by forming cross-links, which prevent cell division, induce apoptosis, inhibit transcription, and disrupt protein synthesis [7]. Cells that are rapidly dividing are especially vulnerable to this process due to their limited time for DNA repair [8]. However, cancer cells can develop resistance to alkylating agents by developing ways to repair DNA damage.

A notable example is temozolomide (TMZ), an alkylating agent widely used for the treatment of glioblastoma multiforme (GBM). However, at least 50% of the patients treated with TMZ do not respond to the treatment [9]. TMZ cytotoxicity is moderated by methylation of the guanine at the N7 position and O6 in genomic DNA; subsequent alkylation of the O6 site during DNA replication is responsible for cell death. It has been demonstrated that many cancer cell lines develop resistance to TMZ due to varying levels of expression of O6-methylguanine-DNA-methyltransferase (MGMT) [10]. In addition, it has also been suggested that another mechanism behind TMZ resistance could be related to the hypomethylation of the MGMT promoter [10]. MGMT is a suicide protein that removes the methyl group from the O6 position of guanine, thereby regenerating guanine after TMZ methylation [11]. Glioblastoma cell lines that express high levels of MGMT are resistant to TMZ, highlighting the need to better understand and overcome resistance mechanisms in cancer therapy [12]. Cells are much less able to develop resistance to the actions of duocarmycin stable A (DSA), which appends the entire molecule to the DNA, than to TMZ, which appends only a small and easily removed methyl group [5,9]. The remarkable potency of DSA against aggressive cancer cell lines, such as GBM, emphasizes the importance of developing alternative alkylating agents that can overcome resistance mechanisms and enhance treatment efficacy.

One of the significant limitations of cancer treatments is tumor heterogeneity. The presence of diverse cell types within the same tumor presents a formidable challenge [13]. First, obtaining a biopsy from a patient is often insufficient for comprehensively understanding the tumor’s genomic landscape due to its cellular heterogeneity [14]. Moreover, this diversity poses challenges as different populations within the tumor may exhibit varying levels of resistance to alkylating agents. Consequently, a single-agent treatment approach may prove effective against one subset of the tumor population but ineffective against another. Therefore, combination therapy emerges as a promising strategy to surmount the challenges posed by the heterogeneous nature of cancer.

For this reason, duocarmycin should be considered as a cancer treatment in combination with other modalities. One way duocarmycin can be integrated into combined therapies is through the addition of radiation. Combining duocarmycin as a synergistic agent with radiation can sensitize radio-resistant cells to radiation by arresting cells in the G2 phase of the cell cycle, which is the most sensitive phase to radiation [15]. As we report in this review, duocarmycin induces G2 arrest in many different cancer types. Thus, administering duocarmycin before radiation may be a powerful approach to increasing the cytotoxic effects of the radiation. This combined treatment strategy has the potential to be effective against different cell populations within a heterogeneous tumor, thereby improving overall treatment efficacy.

One of the primary goals of this review is to provide a comprehensive reference on the biological effects of duocarmycin analogs, serving as a starting point for researchers interested in investigating this class of drugs. While numerous papers have been published on the chemistry of duocarmycin [16,17,18], there is a notable lack of biological studies exploring its effects across different cell lines and elucidating its mechanisms of action. This review aims to detail all biological studies conducted on duocarmycin family members to date. Such a detailed compilation will facilitate a better understanding of duocarmycins’ biological activity and their potential therapeutic applications.

By consolidating the available data, we hope to bridge the gap between chemical synthesis and biological efficacy, thereby enhancing the utility of duocarmycin analogs in cancer research. This comprehensive review will not only highlight the potent cytotoxic effects of duocarmycin analogs, but also underscore the need for further biological investigations to fully harness the therapeutic potential of duocarmycins. All reported in vitro studies accentuate the toxicity of duocarmycin family members, serving as proof of concept. This toxicity is not specific to cancer cells; therefore, targeting duocarmycin analogs is crucial. Current research predominantly focuses on utilizing antibody–drug conjugates with duocarmycin analogs. This review will provide a comprehensive overview of clinical studies and therapeutic applications of duocarmycin and its derivatives.

## 2. Discovery and Development

### 2.1. Historical Background of Duocarmycin Discovery

The discovery and development of the duocarmycins provides a fascinating glimpse into the integration of natural product research and synthetic chemistry, with the goal of finding new and more effective cancer treatments.

**Figure 1:**
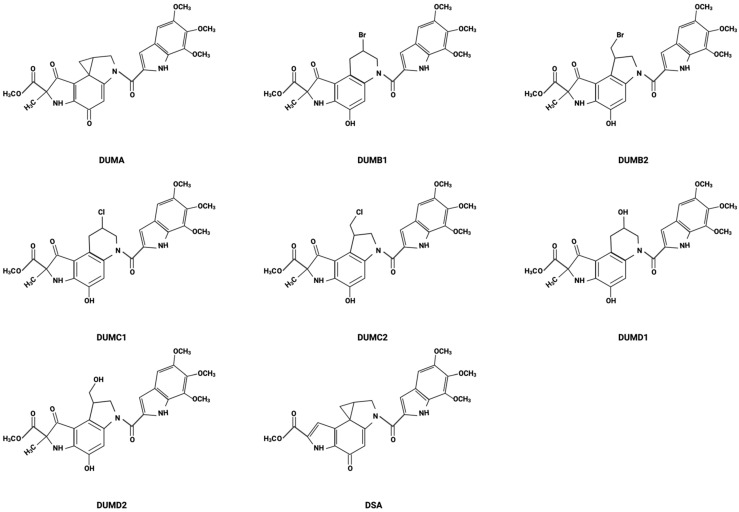
Chemical structures of DSA and its analogs.

The history of DSA begins with the discovery of the duocarmycins, which were first isolated in the late 1980s from the bacterium *Streptomyces* [19]. In 1988, duocarmycin A (DUMA) was isolated from *Streptomyces* DO-88 collected from the foot of Mt. Fuji [20]. Following the discovery of DUMA, researchers were intrigued by its potent cytotoxicity and began to delve deeper into the chemical structure and biological activities of the duocarmycin family. This led to the identification of various analogs, each with unique structural variations that could potentially improve their therapeutic activity (Figure 1). Duocarmycin B1 (DUMB1), duocarmycin B2 (DUMB2), duocarmycin C1 (DUMC1), duocarmycin C2 (DUMC2), duocarmycin D1 (DUMD1), and duocarmycin D2 (DUMD2) were all isolated from *Streptomyces* DO-89 collected from Hyogo, Japan [20]. DSA is the most recently isolated and most potent of the duocarmycin family [19]. DSA was discovered in 1990 and isolated from a strain of *Streptomyces* DO-113 (FERM BP 2222) collected from the soil near the Rokkakudo temple in Kyoto, Japan [19,20,21].

These discoveries were part of a broader effort to explore soil bacteria for novel bioactive compounds with potential medical applications [22]. The initial isolation of duocarmycins was reported by Japanese researchers who were screening for new anticancer antibiotics [19,21]. These compounds immediately attracted attention due to their potent cytotoxicity against various cancer cell lines, even at sub-nanomolar concentrations [23,24]. That these compounds were isolated from such specific and pristine locations suggests that the natural ecological conditions might play a role in the biosynthesis of these complex molecules. It has also furthered the idea of bioprospecting in similar undisturbed environments, reinforcing the idea that biodiversity within such ecosystems is important and perhaps even critical for the discovery of new drugs [22].

### 2.2. Isolation Details

Duocarmycins are a family of antitumor antibiotics known for their potent cytotoxic activities [25]. They were initially discovered from actinomycetes, specifically from strains of *Streptomyces* [19]. The source organism for duocarmycin A was found to produce a unique set of compounds that exhibited highly potent antitumor properties [26,27,28]. The process of isolating duocarmycins generally involves several key steps: collection of soil samples, cultivation of microorganisms, extraction and purification, structural characterization, and biological testing [29]. The first step involves collecting soil samples from various environments. In the case of DUMA, sample collection was performed at the foot of Mt. Fuji, a location that may offer unique biodiversity contributing to the presence of rare microbial species. After collection, soil samples are cultured under various conditions to promote the growth of actinomycetes, specifically *Streptomyces*. These conditions are optimized to encourage the production of secondary metabolites, which often include bioactive compounds. Once the cultures have grown, the secondary metabolites are extracted from the culture medium using solvent extraction techniques. This is followed by various purification steps, such as chromatography, to isolate individual compounds based on their chemical properties. The isolated compounds are then analyzed using techniques such as mass spectrometry (MS) and nuclear magnetic resonance (NMR) spectroscopy to determine their molecular structures, and then, the bioactivity of the compounds, particularly their cytotoxic effects against cancer cells, is tested to identify potential therapeutic applications.

### 2.3. Chemical Structure Elucidation

**Figure 2:**
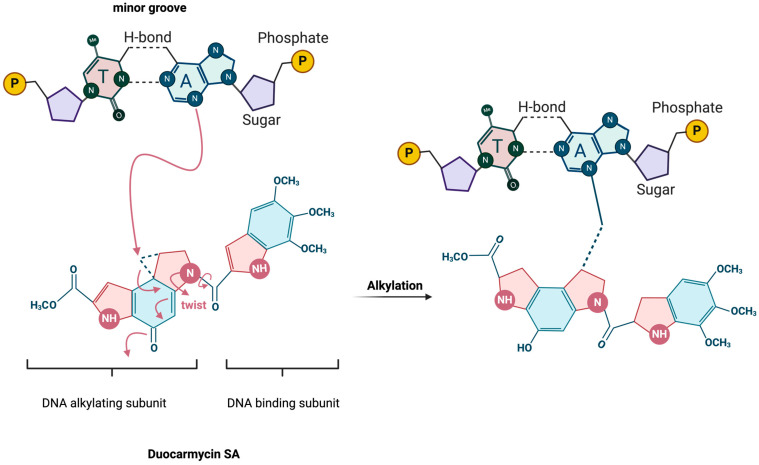
Schematic of DSA-binding and alkylation mechanism. Adapted from Sakata et al. 2019 [33].

The duocarmycin family of compounds are characterized by their ability to bind within the minor groove of DNA (the shape-dependent catalysis indicated by the “twist” in the figure) and alkylate adenine residues, as illustrated by DSA (Figure 2). However, each of these analogs has variations in their molecular structures that confer different degrees of stability, reactivity, or specificity. These variations often occur in the DNA-binding unit, a subunit-linking amide, or the alkylating unit, which significantly influence their biological activity and therapeutic potential [24,30,31,32].

The stability of the different duocarmycins has been studied in relation to their cytotoxicity and antimicrobial activities [34]. These compounds include duocarmycin A and SA, which have a spirocyclopropylhexadienone moiety, and four halogenated seco-compounds of duocarmycin A: duocarmycin B1, B2, C1, and C2, from which the cyclopropane ring structure is absent. The cytotoxic activity was investigated using Balb 3T3/H-Ras cells after 72 h drug exposure. Results showed that duocarmycin analogs induced cytotoxicity, as determined by inhibitory concentrations that inhibited 50% cell growth (IC_50_ (nM)) as follows: DSA (0.05) > DUMA (0.3) > DUMB2 (1.5) > DUMB1 (3.0) > DUMC2 (20) > DUMC1 (40) [34].

Stability studies showed a large difference between DSA and DUMA in aqueous solvents [34]. For the four halogenated seco-compounds, a good correlation was found between their cytotoxicity in vitro and their conversion rate to DUMA, suggesting that halogenated seco-compounds undergo closure to the spirocyclopropylhexadienone structure, the compounds’ most active form, in cells [34].

DSA is representative, though exceptionally potent, within the duocarmycin family due to its structure and unique alkylation properties. Structurally, DSA comprises a three-unit assembly: a DNA-binding unit that ensures specificity to DNA [31], a subunit-linking amide that assists in orienting the alkylating unit [30,32], and the alkylating unit itself, which is critical for its cytotoxic activity [24]. The alkylating unit of DSA reacts selectively within the minor groove of DNA, forming a covalent adduct with adenine bases, which distinguishes it from other alkylating agents that typically target guanine [23,35]. This DNA-specific interaction defines the precision of DSA in targeting DNA and contributes to its efficacy as a potent antitumor agent. DSA exclusively alkylates DNA, while sparing other cellular nucleophiles, due to its unique shape-dependent catalysis mechanism of action [36].

The decades-long exploration of DSA’s potential for incorporation into therapeutic regimens highlights the ongoing pressure to innovate in drug design and development to improve outcomes for cancer patients. Understanding the structural intricacies and biochemical properties of such compounds is crucial for harnessing their full potential, underscoring the significance of structural analysis in advancing cancer therapy.

## 3. Mechanism of Action

### 3.1. DNA-Binding and Alkylation Process

Duocarmycins, including DSA and duocarmycin A (DUMA), represent a highly potent category of DNA-interactive agents that exert their cytotoxic effects via a mechanism of sequence-selective alkylation of duplex DNA [5]. These compounds have demonstrated exceptional efficacy in targeting and modifying the DNA structure, leading to significant antitumor activity.

The molecular structure of duocarmycins, notably DSA and DUMA, features a cyclopropane ring, which is critical for their interaction with DNA [3]. This ring binds within the minor groove of DNA, a narrower and less accessible region that provides a docking site for small molecules [37]. The specificity of this interaction is largely dictated by the molecular configuration of the duocarmycins, which allows them to fit snugly into the minor groove, aligning precisely with the DNA contours [38]. Once bound to the DNA, duocarmycins alkylate adenine residues at the N3 position [23,25,27]. This alkylation is particularly significant as it occurs predominantly at AT-rich regions within the DNA sequence [23]. Alkylating specific regions between nucleosomes can activate endonucleases, leading to heterochromatin condensation and degradation of genomic DNA [39]. The AT-rich areas, having less thermal stability due to the weaker hydrogen bonding between adenine and thymine compared to cytosine and guanine, are more accessible and, thus, more susceptible to interaction with these agents.

### 3.2. Selectivity for Adenine Residues and AT-Rich Sequences

The alkylation process itself is facilitated by a nucleophilic attack from the DNA on the least substituted carbon of the cyclopropane ring [37]. This reaction results in the formation of a covalent bond between the cyclopropane ring of the duocarmycin molecule and the adenine base (Figure 2) [30]. This covalent bonding is irreversible and induces significant conformational changes leading to disruption of normal base pairing in the helical structure and bending or unwinding of the DNA helix. These conformational changes interfere with critical cellular processes such as replication and transcription, pathways especially important during tumor progression [40,41,42,43]. The specificity of the alkylation reaction is further refined by the inherent sequence preference of duocarmycins [23,25,38,40,44]. Research has shown that DSA exhibits a high selectivity for adenine residues that follow two 5′-A or -T bases, with a pronounced affinity for the 5′-AAA sequence [38]. This specificity is influenced by the electronic and steric properties of the surrounding bases, which affect the accessibility and reactivity of the target adenine. The sequence preference extends to a lesser extent to other sequences like 5′-TTA, 5′-TAA, and 5′-ATA, with a general trend showing greater binding affinity when the fourth base of the 5′ sequence is also A or T [45]. To the best of our knowledge, there is no enzyme-mediated reaction that can successfully remove DSA from the target adenine, making this reaction virtually irreversible.

### 3.3. Biochemical Pathways Affected by Duocarmycin-Induced DNA Damage

The alkylation of DNA by duocarmycins triggers a cascade of cellular responses, primarily activating pathways involved in DNA damage recognition and repair [41,46,47]. However, due to the stability and nature of the adducts formed, the DNA damage induced by duocarmycins often proves lethal to cells, particularly cancer cells that have compromised DNA repair mechanisms [46,47]. This makes duocarmycins potent antitumor agents capable of targeting rapidly dividing cancer cells more effectively than normal cells.

## 4. Biological Activities

### 4.1. Cytotoxic Effects of DSA in Various Cancer Cell Lines

DSA and its derivatives are distinguished by their capacity to induce proliferation arrest in cancer cells at picomolar concentrations [5], highlighting their potential as potent therapeutics in the treatment of aggressive cancers. These compounds exert their effects through intricate cell death pathways and have shown efficacy across a range of cancer cell lines, including leukemias and solid tumors, including pancreatic, renal, and prostate cancers.

A study investigated the growth-inhibitory activity of various duocarmycin family members in the human uterine cervix carcinoma cell line, HeLa S_3_. HeLa S_3_ cells were incubated for 1 h with DUMA, DUMB1, DUMB2, DUMC1, and DUMC2. The study reported a concentration-dependent cytotoxicity with IC_50_ values of 0.006 nM, 0.035 nM, 0.1 nM, 8.5 nM, and 0.57 nM, respectively [28]. Notably, compared to its derivatives, DSA exhibited the highest potency in HeLa S_3_ cells, with an IC_50_ of 0.00069 nM [48]. This superior efficacy of DSA was not specific to HeLa S_3_ cells, but common across many cancer types (Table 1).

In one of the first biological studies investigating the duocarmycin family, back in 1994, researchers started exploring the potential cytotoxic effects of DSA on cancer cells [39]. Its findings showed that when cancer cells were exposed to different derivatives of the duocarmycin family with varying concentrations, they underwent apoptotic cell death. This early study shed light on the promising therapeutic potential of the duocarmycin compounds from a biological perspective.

This study also reported the exceptional potency of DSA in different tumor cell lines, including leukemias and pancreatic, renal, and prostate cancers, revealing a remarkable mean IC_50_ of 0.1 nM [39]. Among the various cell lines studied, Molt-4 leukemia cells exhibited blebbing while maintaining cell membrane integrity under the microscope, and treatment with 1 nM of DSA for 4 h resulted in an average 7.6-fold increase in DNA fragmentation compared to the untreated control, indicative of apoptotic cell death. [39]. These findings underscored the potency of DSA in targeting cancer cells at the molecular level.

Moreover, this study is among the few that have investigated the kinetics of DSA uptake by tumor cells. It has been reported that 20 min of incubation allows sufficient DSA uptake by tumor cells; however, a significant decrease in cell viability is observed after an additional 5 h, reaching a threshold by the 20 h mark [39]. In summary, exposure to DSA for 20 min induces an effect, but a minimum of 5 h of incubation is necessary to observe a significant decrease in viability. This study laid the foundation for further research and exploration into the biological aspects of DSA’s potential as a therapeutic agent against cancer cells. It emphasized the importance of understanding its effect beyond just chemical interactions, opening new avenues for investigation into its broader biological mechanisms of action.

### 4.2. Exploring the Effects of DUMA on Human Lung Carcinoma

Studies investigating the effects of DUMA on human lung carcinoma (HLC-2) cells have revealed important insights into its apoptotic mechanisms. The use of annexin-V/propidium iodide (PI) staining demonstrated a dose-dependent increase in both early and late apoptotic events following DUMA treatment. Specifically, DUMA induced significant annexin-V^+^/PI^+^ at 0.003 μg/mL, with further enhancement at 0.03 μg/mL, highlighting its potent apoptotic effects [51].

In parallel, Western blot analysis elucidated alterations in key apoptotic proteins such as caspase-3, caspase-9, Bax, and Bcl-2 in response to DUMA treatment, which are pivotal in the intrinsic apoptosis pathway. The intrinsic apoptosis pathway is initiated by phosphorylation of p53 via Ataxia Telangiectasia Mutated (ATM) and Checkpoint Kinase 2 (Chk2), leading to increased Bax expression and subsequent caspase-9 activation, culminating in caspase-3-mediated apoptotic cell death [51]. Notably, treatment with 0.03 μg/mL DUMA reduced caspase-3 and caspase-9 precursor levels while enhancing Bax expression, underscoring its ability to induce apoptosis effectively [51]. Moreover, cell cycle analysis revealed a pronounced accumulation of cells in the G1 phase upon DUMA treatment, suggesting an additional mechanism for inhibiting cancer cell growth [51].

Collectively, these data support that DUMA treatment of HLC-2 cells induces cell death through DNA damage, leading to the activation of the ATM-Chk2-p53 pathway. Furthermore, intrinsic apoptosis, leading to mitochondrial dysfunction, is associated with increased expression of Bax and cleavage of caspases -9 and -3. Understanding the mechanistic and therapeutic implications of DUMA treatment provides additional insight into its potential therapeutic efficacy, and possible potentiation of treatment effectiveness through targeting components of the pathway (i.e., enhancing ATM activity, stabilizing p53, or mimicking Bax function). Seeing DUMA’s ability to induce phosphorylation of p53, increase Bax expression, and affect caspase levels could serve as biomarkers to predict and monitor treatment responses. These findings collectively highlight DUMA’s potential as an inducer of apoptosis through the intrinsic pathway, making it a promising candidate for therapeutic strategies targeting various cancer cell lines.

### 4.3. Comparison of Duocarmycin A with Another Duocarmycin Derivative

The biological effects of DUMA and its derivative DU-86 were investigated in HeLa S3 cells, focusing on their short-term and long-term impacts through various assays. DU-86 and DUMA differ structurally in terms of the replacement of the pyrrolidone in DUMA with pyrrole in DU-86 [49]. Initially, a growth-inhibitory assay revealed that both compounds induced a concentration-dependent decrease in cell growth, with DUMA demonstrating slightly greater potency than DU-86. The IC_50_ values for DUMA and DU-86 were measured at 0.12 nM and 0.23 nM, respectively. Subsequently, a colony formation assay confirmed these findings, showing that both compounds significantly reduced colony formation in a dose-dependent manner, with DUMA again exhibiting higher potency [49].

Further investigation into the cell cycle phase distribution following treatment with DUMA and DU-86 revealed an S phase arrest after 8 h, persisting longer with DU-86 compared to DUMA. This observation suggests distinct temporal effects on cell cycle progression between the two compounds. Moreover, analysis of DNA damage using pulse-field gel electrophoresis demonstrated that while DUMA induced DNA fragmentation at concentrations of 1.2 nM and higher, DU-86 did not induce DNA strand breaks even at concentrations up to 230 nM. This difference suggests that DU-86 exerts cytotoxic effects through mechanisms other than DNA fragmentation [49].

Overall, these findings indicate the differential potency and mechanisms of action between DUMA and DU-86. DUMA’s superior potency and ability to induce DNA damage, facilitated by its pyrrolidone ring forming adducts with guanine, distinguish it from DU-86. The absence of DNA strand breaks in DU-86-treated cells suggests that this compound uses alternative pathways to exert its cytotoxic effects. These could involve mitochondrial pathways, cell cycle arrest, oxidative stress, interference with cellular signaling, inhibition of DNA repair mechanisms, or disruption of the cytoskeleton. Understanding these alternative mechanisms is essential for fully elucidating the compound’s mode of action and optimizing its use in cancer therapy.

### 4.4. The Role of Reactive Oxygen Species in DUMA-Induced Apoptosis in Human Leukemia Cell Lines

DUMA’s ability to induce apoptosis and the role of reactive oxygen species, specifically H_2_O_2_, in DUMA-induced apoptosis using human leukemia cell lines HL-60 and HP100 was undertaken. The HP100 cell line is an H_2_O_2_-resistant cell line derived from HL-60 through multiple exposures to H_2_O_2_ and passaging of surviving cells. Both cell lines were treated with 10 nM of DUMA for 30 min, 1 h, 2 h, 3 h, and 4 h. DNA fragments and ladder formation were detected using conventional electrophoresis. DUMA induced DNA fragments at 30 min in both HL-60 and HP100 cells, but DNA ladder formation was observed at 2 h only in HL-60 cells. In HP100 cells, DNA ladder formation was observed only after 3–4 h of DUMA treatment [52].

Caspase-1 and caspase-3 activity were also measured using a fluorometric assay. HL-60 and HP100 cells were treated with 10 nM DUMA for 1 h, 2 h, 3 h, and 4 h. In the H_2_O_2_-sensitive cell line HL-60, maximal caspase-3 activity was observed at 3 h, while in the H_2_O_2_-resistant cell line HP100, maximal caspase-3 activity was observed at 4 h. Caspase-1 activity was not detected in either cell line in response to DUMA treatment at the investigated time points [52]. This study elucidates the differential response of H_2_O_2_-sensitive and H_2_O_2_-resistant cell lines to DUMA and underscores the importance of reactive oxygen species in mediating DUMA-induced apoptosis. The findings highlight the potential of targeting oxidative stress pathways in leukemia treatment and provide valuable insights into the mechanisms underlying DUMA’s cytotoxic effects.

### 4.5. Molecular and Cellular Effects of DSA on AML Cells

The most recent study on DSA focused on investigating its biological effects in acute myeloid leukemia (AML) cells [50]. This research was unique for its comprehensive exploration of multiple biological endpoints in response to DSA, utilizing RNA sequencing to identify differentially expressed genes and comparing responses between resistant (HL-60) and sensitive (Molm-14) AML cell lines. Notably, this study represents a pioneering effort in utilizing bulk RNA sequencing to establish a mechanistic model for differential DSA cytotoxicity in resistant and sensitive cell lines, moving beyond a purely phenomenological approach.

In both Molm-14 and HL-60 cell lines, DSA demonstrated considerable efficacy at picomolar concentrations, with IC_50_ values of 11.12 pM and 122.7 pM, respectively, in MTT (3-{4,5-dimethylthiazol-2-yl}-2,5) diphenyl tetrazolium bromide) assays. The study also confirmed the hypothesis that DSA induces DNA damage in the form of double strand breaks. Using γ-H2AX assay, the study revealed a significant increase in γ-H2AX Foci formation in response to picomolar concentrations of DSA in both cell lines [50].

Moreover, the study explored the downstream effects of DSA-induced DNA damage on cell cycle dynamics. Treatment with DSA resulted in G2/M phase arrest in both Molm-14 and HL-60 cells, with an additional increase in the G0 population at higher concentrations, indicative of apoptotic cell death [50]. Subsequent analysis using annexin-V and -PI staining via flow cytometry revealed that high concentrations of DSA triggered apoptosis in both cell lines [50]. Specifically, concentrations above 100 pM resulted in more than 50% of Molm-14 cells undergoing apoptosis, while concentrations of 250 pM and higher induced apoptosis in more than 50% of HL-60 cells. The dose-dependent increase in apoptotic cells, especially at higher concentrations, underscores the effectiveness of DSA in inducing programmed cell death. These findings assert DSA’s potential as a powerful chemotherapeutic agent, particularly for cancers characterized by rapid cell division and resistance to conventional therapies. Further research should focus on elucidating the detailed molecular mechanisms by which DSA induces these effects and exploring its efficacy in combination with other treatments to enhance therapeutic outcomes.

RNA-sequencing analysis revealed differential expression of DNA repair genes (RFC5, RFC2, PCNA, RPA2, RAD51, FEN1) in resistant HL-60 cells compared to sensitive Molm-14 cells post-DSA treatment. The upregulation of DNA repair genes in HL-60 cells suggests that these cells activate an enhanced DNA repair response, contributing to their resistance to DSA. In contrast, the downregulation of these genes in Molm-14 cells indicates a compromised repair capability, making them more susceptible to DSA-induced apoptosis [50]. These findings highlight the importance of DNA repair pathways in mediating resistance to chemotherapeutic agents and underscore the potential of targeting these pathways to improve therapeutic efficacy in resistant cancer cells.

This study provides a comprehensive insight into the mechanisms underlying DSA cytotoxicity in AML cells, highlighting the roles of DNA damage induction, cell cycle arrest, apoptosis, and differential DNA repair gene expression. These findings contribute significantly to our understanding of DSA’s therapeutic potential in AML treatment, suggesting the importance of targeting DNA repair mechanisms to enhance therapeutic efficacy. Future research should explore strategies to inhibit DNA repair mechanisms in resistant cells like HL-60 to enhance the effectiveness of DSA and similar cytotoxic agents. Additionally, understanding the specific pathways and factors involved in the differential gene expression could provide new targets for overcoming drug resistance in cancer therapy and lead to the development of more effective strategies for treating AML.

### 4.6. DSA and Proton Radiation Combination Therapy in GBM

In a 2018 study, with the intent to explore new treatment modalities for the treatment of glioblastoma multiforme, the authors investigated the effects of DSA alone and in combination with proton radiation on the GBM cell line U-138, providing an initial assessment through colony formation, cell proliferation, and apoptosis/necrosis assays [5]. The study revealed potent activity for DSA alone against U-138 with an IC_50_ of 0.0018 nM in the clonogenic assay conducted over 14 days. Short-term effects on viability were further investigated with varying concentrations of DSA, demonstrating an IC_50_ of 0.4 nM on cell proliferation rate [5]. These results accentuate the strong cytotoxic potential of DSA as a standalone treatment, capable of effectively reducing cell viability and colony formation at sub-nanomolar concentrations. Moreover, when DSA was combined with proton radiation at a dose of 2 Gy, a modest additive effect was observed. The combination of 0.1 nM DSA with 2 Gy of protons resulted in a dose enhancement ratio of 1.6, indicating an enhanced therapeutic effect compared to either treatment alone [5].

The study demonstrates that DSA is a highly potent antitumor agent against the GBM cell line U-138, both as a standalone treatment and in combination with proton radiation. The significant reduction in cell viability and colony formation at low concentrations of DSA highlights its potential effectiveness. Additionally, the combination with proton radiation, although showing a modest additive effect, suggests that DSA can enhance the overall therapeutic outcome, potentially offering a synergistic approach to improve GBM treatment efficacy.

The study assessed apoptosis and necrosis by measuring annexin-V- and PI-positive cells, in U-138 cells treated with DSA alone and in combination with protons. Treatment of cells with 0.1 nM DSA showed approximately 30% annexin-V positivity, compared to approximately 10% in the control group, reiterating DSA’s ability to induce apoptotic cell death. Importantly, the treatment also induced less than 10% necrotic cell death, indicating specificity towards apoptotic pathways [5]. Further analysis revealed that the combination of 0.1 nM DSA with 2 Gy of proton radiation resulted in an additive effect on both apoptosis and necrosis. Specifically, the combination therapy exhibited a dose enhancement ratio of 2.5-fold for apoptosis and 4 for necrosis, suggesting enhanced cellular damage [5].

This study provides insights into the therapeutic potential of DSA in GBM treatment when combined with proton radiation. These findings highlight DSA’s ability to inhibit colony formation, reduce cell proliferation, and induce apoptosis, thereby enhancing treatment outcomes in GBM. Future research would benefit from the elucidation of the underlying molecular mechanisms and optimization of therapeutic strategies to maximize the clinical benefits of DSA and proton radiation combination therapy in GBM patients.

### 4.7. Targeting Senescent Cancer Cells

Recent studies have focused on investigating the effectiveness of duocarmycin against senescent cancer cells, highlighting a novel approach in cancer therapy. Senescence, while initially considered beneficial for its inhibition of cell proliferation, poses a challenge as senescent cancer cells can re-enter the cell cycle and contribute to tumor repopulation [53]. In 2021, a significant advancement was made with the development of the first senolytic antibody–drug conjugate (ADC) targeting senescence, conjugating the senescence marker B2M with duocarmycin (B2M-ADC). B2M-ADC was tested on human bladder carcinoma cell lines (EJp53) and a colorectal carcinoma cell line (HCT116) with tet-regulatable p53 [54]. The study aimed to leverage ADC technology to selectively target the stress-induced p53-dependent senescent phenotype using duocarmycin [54]. Results demonstrated that the B2M-ADC conjugate was effectively internalized into senescent cells, leading to a concentration-dependent decrease in cell viability. Furthermore, subsequent cell death analysis revealed an increase in necrotic cell death in EJp53 cells treated with B2M-ADC, while apoptosis levels remained comparable to controls. Importantly, the antibody specifically targeted senescent cells without affecting non-senescent cells, validating the treatment’s specificity [54].

Another innovative approach involved the use of galactose-modified duocarmycin (GMD) to selectively eliminate senescent cells [55]. GMD is a prodrug that has been chemically modified to include a galactose moiety. This modification targets the prodrug to senescent cells, which are characterized by elevated levels of senescence-associated β-galactosidase (SA-β-gal) activity [56]. The SA-β-gal enzyme is prevalent in senescent cells due to increased lysosomal content, a hallmark of cellular senescence. This strategy effectively increased caspase -3/-7 activity specifically in senescent cells, highlighting duocarmycin’s potential in targeting and eliminating this distinct population of cells [55]. These studies point out the novelty and significance of utilizing duocarmycin in combating senescent cancer cells. By specifically targeting senescent cells through B2M and galactose modification, duocarmycin demonstrates promise in addressing the challenges posed by senescence-associated tumor repopulation. These findings not only advance our understanding of senolytic therapies but also pave the way for future research aiming to refine and optimize duocarmycin-based treatments for cancer therapy.

## 5. Preclinical Studies

The journey from discovery to clinical application of new antitumor agents is a complex and rigorous process involving multiple phases of research and testing. This process begins with the identification and initial evaluation of compounds for their cytotoxic properties. While early studies on such compounds often reveal significant antitumor activity, the path to clinical use is fraught with challenges, including toxicity and adverse effects observed in vivo. The ultimate goal of these studies is to develop safe, effective cancer treatments that improve patient outcomes and quality of life. The rigorous process ensures that only the most promising and safe therapies make it to clinical use, contributing to the advancement of oncology and offering new hope for cancer patients.

**Figure 3:**
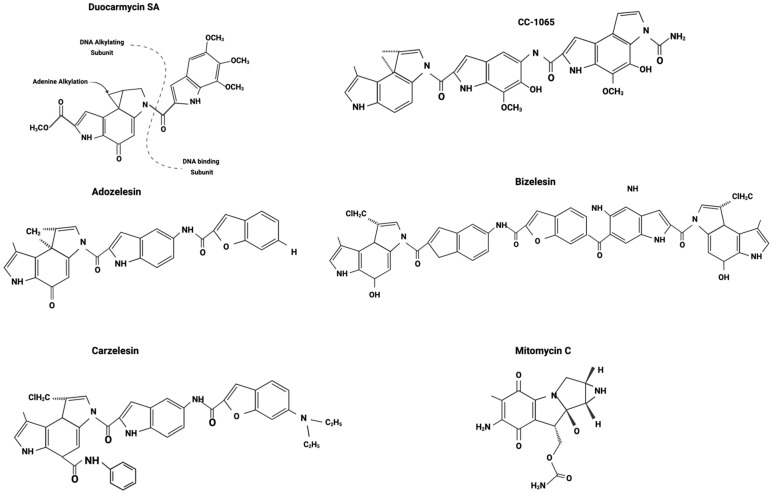
Duocarmycin derivatives. Adapted from Puyo et al. 2014 [35].

Among the eight duocarmycins isolated from *Streptomyces*, three synthetic analogs of CC-1065—adozelesin, carzelesin, and bizelesin—as well as a derivative of duocarmycin B2, pibrozelesin, were studied for their potential to treat cancer from the 1990s to the early 2000s (Figure 3). More recently, yatakemycin, another duocarmycin isolated from *Streptomyces*, has shown potential as a cancer treatment. However, studies on yatakemycin are limited. In this section, we explore the discovery of these drugs and their mechanisms of action from a molecular biology perspective, examining past research and experiments.

### 5.1. CC-1065

In 1978, scientists at The Upjohn Company isolated CC-1065 from the fermentation broths of *Streptomyces zelensis* [57]. CC-1065 was the first duocarmycin to be isolated and evaluated as an antitumor agent. Since the early 1980s, numerous in vitro and in vivo studies have examined the therapeutic potential of CC-1065 in treating cancers.

One of the earliest studies on CC-1065 confirmed its potency, stating that CC-1065 is 400 times more cytotoxic than the anthracycline medication doxorubicin. This conclusion was based on a cell growth inhibition, with CC-1065 showing an ID_90_ of 0.05 ng/mL compared to 20 ng/mL for doxorubicin in L1210 cells during a 72 h in vitro drug treatment [58]. Furthermore, the initial mechanism of DNA alkylation by CC-1065 was identified. It has been shown that CC-1065 strongly interacts with double stranded DNA by binding to adenine- and thymine-rich minor grooves [59,60], and inhibited the activities of thymidine kinase and DNA polymerase alpha, thereby inhibiting DNA synthesis [58]. CC-1065 also demonstrated its effectiveness in inhibiting DNA synthesis; however, it required a high concentration to inhibit RNA synthesis and had little effect on protein synthesis [58,61]. A human tumor-cloning assay examined the lethality of CC-1065 on 15 types of cancer cells. The results indicated varying responses among different cancer types. Melanoma, leukemia, esophageal cancer, lymphoma, colon cancer, and sarcoma showed no response to a 1 h treatment of CC-1065 at 0.1 ng/mL, while the nine other cancer types responded to the drug with a decreased number of colonies. CC-1065 did not cause DNA breaks, but it inhibited the susceptibility of DNA to nuclease S1 digestion [58,59,62].

CC-1065 was able to inhibit DNA synthesis and cell growth at much lower concentrations compared to other cancer drugs, which intrigued scientists, prompting further in vivo experiments to promote clinical trials. One of the in vivo experiments involved transplanting six different tumor types into mice and treating them with CC-1065 via intraperitoneal injections [63]. The results showed that while CC-1065 did not cure cancer, it did prolong the lifespan of the mice. However, a significant in vivo experiment demonstrated that CC-1065 caused death in normal mice and rabbits several days after drug administration [64]. The drug was administered either once or multiple times using different methods, including intravenous and intraperitoneal injections. Mice treated with high intravenous doses died within 12 days, showing hepatic necrosis. Mice treated with lower doses died with changes in hepatic mitochondrial morphology. These deaths were observed at therapeutic antineoplastic doses, leading to the termination of further efforts to utilize CC-1065 for cancer treatment.

### 5.2. Adozelesin (U-73,975)

Captivated by the potency of CC-1065, scientists at The Upjohn Company pursued the creation of synthetic analogs of CC-1065 that were less toxic, leading to the development of adozelesin (U-73,975) and carzelesin (U-80,244). In contrast to CC-1065, adozelesin did not cause delayed death in normal mice [65]. Furthermore, adozelesin effectively reduced tumor weight when administered intravenously against murine tumors, including leukemia, melanoma, sarcoma, colon, pancreas, and Lewis lung carcinomas. Adozelesin was also highly effective against human tumor xenograft models, including colon, lung, clear cell, and ovarian carcinomas. Compared to cisplatin, cyclophosphamide, and doxorubicin, mice treated with adozelesin were more active than those treated with the other drugs.

Ayash et al. examined anticancer drug synergy by combining adozelesin with clinically approved cancer drugs on MCF-7 and MCF-7/CP (cisplatin-resistant subline) using a colony formation assay [66]. The combination of adozelesin and cisplatin (CDDP) showed greater-than-additive, synergistic cytotoxicity in both MCF-7 and MCF-7/CP cells. The combination of adozelesin and carboplatin showed greater-than-additive cytotoxicity in MCF-7 cells and additive cell killing in MCF-7/CP cells. The combination of adozelesin and carmustine (BCNU) exhibited greater-than-additive cytotoxicity in MCF-7/CP cells and additive cell killing in MCF-7 cells. The combination of adozelesin and melphalan (L-PAM) showed greater-than-additive cytotoxicity in MCF-7 cells, whereas in MCF-7/CP cells, the combination of adozelesin and a high concentration of L-PAM led to greater-than-additive effects. The combination of adozelesin and thiotepa showed additive or less-than-additive cytotoxicity in MCF-7 cells but greater-than-additive cytotoxicity in MCF-7/CP cells.

In a colony formation assay, adozelesin demonstrated significantly greater potency against human ovarian cancer cells (A2780) than rodent cells, with an LD_90_ of 0.048 nM. The study also conducted a human tumor-cloning assay using 12 different human cancer types obtained from tumor biopsies. Cells were treated with several drugs for 1 h or continuously, and adozelesin significantly reduced colony formation compared to other drugs, including isophosphamide, vinblastine, doxorubicin, mitomycin C, VP-16, bleomycin, 5-fluorouracil, and methotrexate. The study further analyzed the response of cells at different cell cycle phases to adozelesin using V79, a Chinese hamster cell line. Cells in the M and early G1 phases (0–1 h) were most resistant to adozelesin, while cells in the late G1 (2–3 h) and mid-S (5–6 h) phases were most sensitive, and those in the late S phase displayed intermediate sensitivity. In examining the effects of adozelesin on the cell cycle, three distinct effects were observed: adozelesin slowed the progression of the S phase, blocked the G2-M phase for about 32 h, and caused cells that escaped the G2-M block to enter a second round of DNA synthesis without undergoing cytokinesis, resulting in polyploidy. Additionally, adozelesin, similarly to CC-1065, inhibited DNA synthesis at a much lower concentration than it inhibited RNA or protein synthesis. The concentration of adozelesin required for 50% growth inhibition was 10 times lower than the concentration required for 50% inhibition of DNA synthesis. Research demonstrating adozelesin’s significant effects on cell cycle blocks prompted further investigation into its impact on five different gynecologic cancer cell lines [61,67]. Although each cell line exhibited different sensitivity to adozelesin and the cell cycle blocks were dose-dependent, adozelesin primarily induced cell cycle arrest in the S and G2 phases.

To examine the mechanism by which adozelesin inhibits DNA replication and induces DNA breaks, this study used yeast cells with checkpoint kinase mutations [68]. RAD53 and MEC1, equivalent to human CHK2 and ATM, regulate a checkpoint during the S phase of cell division that ensures DNA damage is repaired before replication continues. The results showed that adozelesin inhibited the activity of active replication origins and fork progression despite the mutations in RAD53 and MEC1, confirming its ability to alkylate DNA. However, adozelesin also activated normally silent replication origins on the same chromosome, causing abnormal replication patterns in RAD53 and MEC1 mutated cells. This is an important finding, as it shows that while adozelesin damaged DNA, it also induced abnormal replication. Additionally, DNA breaks were only detected in RAD53 mutant cells.

This study confirmed the effects of adozelesin on DNA damage responses in the S phase of the cell cycle [69]. Adozelesin exerts a cytotoxic effect, like DSA, by binding to the minor groove of DNA and forming DNA adducts. This inhibition triggers the hyperphosphorylation of Replication Protein A (RPA) and the formation of RPA and γ-H2AX foci, indicating DNA damage. These responses are dependent on active DNA replication, as evidenced by the sensitivity to aphidicolin, a replication inhibitor that blocks RPA and γ-H2AX focalization. Unlike other DNA-damaging agents that cause direct DNA strand breaks, adozelesin requires replication fork progression to induce damage signals. Because adozelesin treatment does not directly induce DNA breaks, the study concluded that adozelesin induces S phase-specific DNA damage and that the S phase cell cycle arrest is triggered by stalled replication forks rather than DNA strand breaks.

The major limitation of adozelesin has been identified in an in vivo study, which demonstrated that adozelesin significantly reduced tumors in mice with human lung LX-1 or advanced-stage human ovarian 2780 carcinoma [70]. However, tumor regrowth recurred and surpassed primary tumor volume within 40 days of ending adozelesin treatment. This indicates a major limitation in the longevity of adozelesin treatment in vivo.

### 5.3. Carzelesin (U-80,244)

Carzelesin is a prodrug and synthetic analog of CC-1065. The activation of carzelesin involves two steps: first, the hydrolysis of a phenylurethane group to produce U-76,073, and second, the ring closure that results in the formation of the cyclopropyl-containing DNA-reactive U-76,074. Based on in vitro experiments conducted on 10 different gynecologic cancer cell lines, which compared adozelesin, carzelesin, and bizelesin, adozelesin demonstrated the highest potency, being 103 to 104 times more cytotoxic than cisplatin and adriamycin [71]. Bizelesin and carzelesin were less potent than adozelesin but exhibited 100 to 1000 times greater cytotoxicity than cisplatin and adriamycin. Based on an ATP-based tumor chemosensitivity assay, 100% of the cell lines were sensitive to adozelesin, 80% were sensitive to bizelesin, 40% were sensitive to carzelesin, 16% were sensitive to cisplatin, and 33% were sensitive to adriamycin. Although carzelesin was significantly more potent than cisplatin, it was the least potent when compared to other CC-1065 analogs.

In an in vivo study, carzelesin showed significant activity against colon tumors and rhabdomyosarcoma xenografts, observed only at the highest nonlethal dose levels [72]. The anticancer effectiveness was assessed by the observed decrease in tumor growth. At the highest dose, carzelesin inhibited growth in four of six colon tumor lines, with one line showing partial regression. For rhabdomyosarcomas, carzelesin administered at the highest nonlethal dose level inhibited tumor growth, with four out of six tumor lines showing partial or complete regressions. In comparison with adozelesin, which showed significant recurrence after cessation of therapy, carzelesin therapy showed significantly less tumor regrowth in mice after cessation [70].

### 5.4. Bizelesin (U-77,779)

Bizelesin is a synthetic analog of CC-1065 and a bifunctional alkylating agent that contains two chloromethyl precursors of the CPI functional group linked by an indole–ureido–indole tether. Bizelesin binds primarily to the T(A/T)4A motif, forming interstrand cross-links that lead to disruption of DNA replication [73].

Preclinical evaluations demonstrated that bizelesin induced antitumor responses across a spectrum of syngeneic murine tumors and human tumor xenografts in mice, including leukemia, melanoma, and renal, colon, and lung cancers [74]. These effects were observed without its causing the delayed deaths noted with its parent compound, CC-1065. One significant finding was that in mice subcutaneously implanted with L1210 leukemia and treated intraperitoneally, 8 out of 10 mice were tumor-free by day 28. The study highlighted that bizelesin achieved at least a 1.0 log10 cell kill (90%) in a broad range of human tumor xenograft models and a 6.7 log10 cell kill (99.9%) against intraperitoneally implanted P388 and L1210 leukemias. It should be noted that this cell kill rate was calculated using a formula presented in the publication, based on the difference in the median day of death between the treated and control groups, rather than by directly confirming cancer cell death.

In V79 Chinese hamster lung cells, researchers induced resistance to bizelesin by continuously exposing the cells to the drug with gradually increasing concentrations [75]. The resistant cells were 125- to 250-fold more resistant than the sensitive cells. These resistant cells exhibited a multidrug-resistant phenotype in overexpressing multidrug-resistant mRNA, and demonstrated cross-resistance to several unrelated drugs, such as colchicine, actinomycin D, and adriamycin. The sensitive cells (V79/S) showed dose-dependent accumulation in the G2-M phase and increased polyploidy at higher concentrations. In contrast, resistant cells (D6) accumulated in the G2-M phase at higher drug concentrations but largely avoided polyploidy. Resistant cells were able to escape the G2-M block and return to normal phase distribution more effectively than sensitive cells. Moreover, synergistic effects were observed in the resistant cell line when treated with verapamil and bizelesin.

Another study investigated the ability of bizelesin to break DNA strands [76]. DNA interstrand cross-links were detected only when cells were exposed to high concentrations of bizelesin for 6 h, and no other forms of DNA damage were observed. DNA interstrand cross-links were found when HT-29 cells were treated with 30–60 pM of bizelesin and BE cells with 20 pM of bizelesin. These concentrations are higher than those required to produce a 3-log cell kill in colony formation assays. At pharmacologically relevant concentrations, no DNA damage, such as singlestranded breaks, interstrand cross-links, or DNA–protein cross-links, was detected in either cell line.

A subsequent study conducted by the same author showed that bizelesin-induced DNA interstrand cross-links are reversible upon exposure to alkaline conditions [77]. The study demonstrated that the covalent DNA adducts undergo two competing reactions: depurination leading to DNA strand cleavage and adduct reversal restoring DNA integrity. Three factors have been identified to cause adduct reversal: high pH (alkaline conditions), elevated temperature, and high ionic strength. The findings suggest that the failure to detect CPI-induced DNA adducts in cellular DNA may be due to the adduct reversal favored under alkaline conditions.

Another study investigated DNA damage response pathways induced by adozelesin and bizelesin in HCT116 cells [78]. The research demonstrated that 0.5 nM concentrations of adozelesin caused a transient S phase block and cell cycle arrest in G2-M, with increased induction of p53 and p21. A 2.5 nM concentration led to apoptosis without p21 induction. On the other hand, bizelesin, at both low and high concentrations, enhanced p53 and p21 induction, triggered G2-M cell cycle arrest, and led to cellular senescence without significant apoptosis. Although adozelesin and bizelesin utilize a similar mechanism to alkylate DNA, they decreased HCT116 tumor cell proliferation through different pathways: adozelesin primarily induced apoptosis, while bizelesin induced senescence.

### 5.5. Pibrozelesin (KW-2189) DAerivative of Duocarmycin B2

One of the limitations of duocarmycins as drug candidates has been their poor water solubility, which led to the introduction in pibrozelesin of a water-soluble functional group consisting of a basic amine incorporated as an enzymatically labile N-methyl piperazine carbamate ester, producing a water-soluble synthetic prodrug analog of duocarmycin B2 [79]. The very first in vitro experiment examining pibrozelesin’s antitumor ability in human small cell lung cancer cells is interesting; it found that pibrozelesin required carboxyl esterase to induce significant cytotoxicity and DNA breaks in the in vitro assay [79]. Moreover, DNA treated with esterase-activated pibrozelesin was protected from digestion by some restriction enzymes. Carboxyl esterase activates pibrozelesin by removing the N-methyl piperazine side chain, allowing for cyclization to produce the cyclopropane ring characteristic of the duocarmycins, which in turn led to DNA alkylation.

Kobayashi et al. conducted in vivo experiments utilizing pibrozelesin [80]. Pibrozelesin significantly inhibited the growth of several murine solid tumors, including Colon 26 adenocarcinoma, Colon 38 adenocarcinoma, and B16 melanoma, as well as murine P388 and L1210 leukemia through both local and systemic administration. Pibrozelesin also demonstrated efficacy against various human xenografts in mice, including tumors from lung, stomach, liver, pancreas, and breast. It showed response in 14 out of 16 tested tumors, including those resistant to other drugs. Pibrozelesin did not cause the delayed lethal toxicity observed in mice treated with CC-1065. Like other duocarmycin analogs, pibrozelesin inhibited DNA synthesis more significantly than RNA or protein synthesis. The study showed that pibrozelesin’s cell growth-inhibitory activity was enhanced by treatment with porcine liver esterase, mouse liver homogenate, or human liver carcinoma Hep G2 homogenate, converting it to DU-86, an active metabolite. When comparing pibrozelesin and DU-86, pibrozelesin was more effective at antitumor activity against a human xenograft model. The study examined the independent abilities of pibrozelesin, DU-86, and duocarmycin B2 to cause DNA strand breaks in vitro. DNA strand breaks were only observed in HeLa S3 cells treated with duocarmycin B2. Duocarmycin B2 was able to break DNA at 0.37 nM to around 1,500 kilobases, the same concentration as its IC_50_ value after 1 h of treatment. Pibrozelesin did not cause DNA strands to break even at a concentration 100 times its IC_50_, which was 38 μM, as expected, given the absence of carboxyl esterase in the assay. The half-life of DU-86 was 2.6 h, and that of pibrozelesin was 20 h in the culture media.

A subsequent study examined the effectiveness of pibrozelesin in cisplatin- and cpt-11-resistant small cell lung cancer and ovarian cancer cell lines [81]. The study found a direct correlation between intracellular carboxyl esterase level and the sensitivity of cancer cell lines to pibrozelesin. It showed that cisplatin-resistant cell lines inherently had lower expression levels of carboxyl esterase compared to sensitive cell lines, making the resistant cell lines also more resistant to pibrozelesin. However, by treating these resistant cell lines with carboxyl esterase extracellularly, they were able to increase the sensitivity to pibrozelesin. Increasing carboxyl esterase in the resistant cell lines did not impact their sensitivity to cisplatin. These findings underscore the key role of carboxyl esterase in activating pibrozelesin and highlight its critical role in the drug’s antitumor activity.

### 5.6. Yatakemycin

Yatakemycin (YTM) is the latest natural duocarmycin to be isolated from *Streptomyces*, which was extracted in 2003 by Kaken Pharmaceutical Co. in Japan [82]. This study further conducted MTT assays in four different cancer cell lines, confirming that YTM was 1,000-fold more cytotoxic than mitomycin C.

Mullins et al. demonstrated that bulky DNA lesions caused by YTM are removed by the nucleotide excision repair pathway and that YTM adducts can also be repaired by the base excision repair pathway through the action of bacterial DNA glycosylases, AlkD (alkylpurine glycosylase D) and YtkR2 [46]. AlkD was shown to remove the yatakemycin-induced lesion from the DNA by prying open the minor groove, disrupting these interactions, and allowing access to the N-glycosidic bond for hydrolysis. However, the DNA repair mechanism had limitations due to the extreme stability of the YTM-DNA substrate and the AlkD/AP-DNA product complex, which inhibits further repair.

The only duocarmycin drugs that have progressed to clinical trials for intravenous cancer treatment are adozelesin, carzelesin, bizelesin, and pibrozelesin (Table 2). However, dose-limiting myelosuppression was observed in all trials, and the overall drug efficacy was not significant. This has led to current efforts in developing antibody–drug conjugates, where duocarmycins are used as payloads.

## 6. Progress of Synthetic Analogs of Duocarmycin in Clinical Trials

The synthetic analogs of duocarmycin, particularly adozelesin, carzelesin, bizelesin, and pibrozelesin, have been tested extensively in clinical trials to evaluate their efficacy and safety in treating various cancers (Table 2). Researchers conducted several Phase I trials with adozelesin involving patients with refractory solid tumors. Despite varying dose schedules, the trials consistently identified myelosuppression as the dose-limiting toxicity, with minimal antitumor responses [83,84,85,86]. In a Phase II trial for untreated metastatic breast cancer, limited efficacy was observed, and the trial was halted early due to slow enrollment and adverse effects, primarily myelosuppression [87].

Carzelesin also demonstrated dose-limiting myelosuppression in multiple Phase I trials, with doses escalated to as high as 300 µg/m^2^. Studies reported partial responses in some patients, yet overall, the drug showed limited efficacy [88,89,90]. Finally, a Phase II trial found carzelesin ineffective in treating colorectal, gastric, melanoma, and other cancers, with myelotoxicity being the most frequent adverse effect [91].

Bizelesin trials similarly highlighted neutropenia as a dose-limiting toxicity. Despite no objective responses in Phase I trials [92,93], a notable reduction in tumor size was observed in a patient with advanced ovarian carcinoma, indicating potential for isolated cases. No Phase II trials have been reported to date, and, thus, bizelesin still holds some promise clinically.

Pibrozelesin, evaluated in both Phase I and II trials, showed hematologic toxicity as the primary limiting factor. Phase trials observed no significant objective responses in metastatic renal cell carcinoma and hepatocellular carcinoma patients [94,95,97]. However, a Phase II trial for metastatic melanoma demonstrated a 17% overall objective response rate, with some patients achieving long-term survival [96].

Overall, these trials underscore the challenges associated with synthetic duocarmycin analogs, primarily due to severe hematologic toxicities and limited antitumor efficacy. Further research is necessary to optimize these compounds for clinical use, potentially through combination therapies or novel delivery mechanisms such as ADCs to mitigate adverse effects, potentiate activity, or direct them to a specific tumor type.

## 7. Duocarmycin-Based Antibody–Drug Conjugates

Antibody–drug conjugates (ADCs) combine the antigen specificity of a monoclonal antibody (mAb) with various chemotherapeutic agents to achieve targeted cancer therapy, potentially changing the pharmacokinetic (PK) and toxicological profile of the chemotherapeutic agent [4,98]. Due to duocarmycin’s cytotoxic potency, and narrow therapeutic window, it represents an excellent compound to integrate in an ADC to target treatment and limit toxicity [4,99].

The efficacy of duocarmycin-based ADCs depends on multiple factors, including therapeutic target selection, mAb specificity, mAb biological activity, and duocarmycin analog selection. It is worth noting that all ADCs that have progressed to clinical trials so far have used a duocarmycin prodrug as a conjugate [4,100,101,102].

An example of the importance of adequate target selection is Promiximab-DUBA, which binds to unknown specific isoforms of CD56 expressed only in some squamous cell lung cancer (SCLC) cell lines, leading to a limitation in performance for CD56-positive tumors with different isoforms [103].

Another relevant treatment strategy is for the mAb component of an ADC to block growth signaling and induce receptor internalization. A model of this strategy is SYD985, a trastuzumab-based ADC that adds the HER-2 receptor blockage and internalization inherent to the mAb with intracellular drug delivery, resulting in high cytotoxicity. SYD985 is the ADC with the most progress in clinical trials to date. SYD985 is currently in a Phase III clinical trial for select breast cancer patients [4]. This ADC contrasts with other conjugates with moderate or unknown mAb biological activity, such as D2B–duocarmycin, intended for prostate cancer, and CyEt-Pan-Duo, targeting EGFR, showing no tumor growth inhibition and growth inhibition without tumor-shrinking, respectively [104,105].

In addition to SYD985, other duocarmycin-based ADCs have progressed to clinical trials, two of which are in Phase I; Syd1875 targeting 5 T4 in colorectal, kidney, or gastrointestinal neoplasms, and Mgc018 targeting B7-H3 for prostate, lung, breast, or other solid tumors [4,106]. However, another clinical trial with Bms-936561 mAb against CD70 was terminated in Phase I because of safety and efficacy concerns [101].

Many of the duocarmycin-based ADCs still have unknown toxicologic and PK profiles, with favorable in vitro cytotoxic results warranting further research [4,107]; novel approaches to duocarmycin-based ADCs include the integration of bispecific mAb with anti-target and an anti-cotinine–duocarmycin domains [108], and the use of duocarmycin-derived dimers [109].

## 8. Conclusions

Adaptive cancer resistance mechanisms necessitate the exploration of potent therapies, such as duocarmycin and its analogs. The duocarmycin family of drugs stand out as promising agents against aggressive cancers by their demonstrated efficacy, shown through the capacity to cause DNA fragmentation and induce distinct morphological changes indicative of programmed cell death in cancerous cells [39]. This cytotoxicity is further enhanced when DSA is combined with proton radiation, underscoring its potential as a component in synergistic treatment modalities [5].

Sub-nanomolar concentrations of DSA can markedly enhance the cytotoxic effects of proton radiation on GBM cell lines [5]. This is demonstrated by significantly reduced cell survival and proliferation rates, highlighting the potential of this combination therapy. While historical toxicity concerns have limited the clinical use of duocarmycins, recent advancements in ADCs have reignited interest in their use, with promising outcomes in early clinical trials. The transformation of duocarmycins into ADCs marks a significant leap forward, as evidenced by Yao et al.’s comprehensive review encompassing various duocarmycin-based ADC candidates [4]. This evolution not only expands the therapeutic options against cancer but also highlights the versatility and adaptability of duocarmycin in therapeutic innovation. Moreover, targeted radiosensitizers like DSA show promise in selectively sensitizing tumor cells to lower radiation doses, potentially widening the therapeutic window and sparing healthy tissue from the toxic effects of standard radiation doses. These advancements highlight the ongoing potential of DSA and similar compounds in enhancing cancer treatment outcomes through innovative therapeutic approaches.

Despite these advancements, there remains a critical gap in understanding the precise mechanisms of DSA’s toxicity. While apoptosis has been identified as a primary mode of cell death in response to DSA treatment, differences between apoptotic cell death rates and overall reductions in cell viability suggest the involvement of supplementary pathways. There is a need to further investigate other modalities of cell death, such as necroptosis and senescence in response to DSA administration. Duocarmycin exhibits cytotoxic effects against senescent cells; however, it is crucial to investigate whether duocarmycin itself triggers cellular senescence. Although senescence is considered a therapeutic approach in cancer due to its proliferation-inhibiting properties, senescent cells can potentially re-enter the cell cycle and contribute to tumor repopulation [53]. Therefore, if future studies reveal that duocarmycin induces senescence, it may be necessary to explore additional modalities to enhance its effectiveness. Alternatively, duocarmycin could be conjugated with senescence markers, as demonstrated in previous studies, to target and eliminate the senescent cell population effectively [54,55].

Future research should focus on elucidating the molecular pathways and key cellular mechanisms responsible for duocarmycin-induced cytotoxicity. A promising approach involves comprehensive proteomics analysis. This methodology aims to identify specific proteins that undergo over- or underexpression in response to duocarmycin derivatives, potentially elucidating key molecular players involved in duocarmycins’ potent cytotoxic effect. Once these critical proteins are identified, drug combination experiments should be designed to inhibit these proteins and assess whether their inhibition affects the potency of duocarmycin derivatives. This strategic approach not only promises to enhance our understanding of the underlying mechanism of duocarmycin’s cytotoxicity, but also holds potential for identifying novel therapeutic targets to optimize the therapeutic potential of duocarmycin-based treatments.

Such insights will be instrumental in overcoming existing challenges in treating resistant cancer and enhancing patient outcomes. By deepening our understanding of these mechanisms, we can effectively harness the full therapeutic potential of DSA-based therapies and pave the way for more targeted and personalized cancer treatments in the future.

## Figures and Tables

**Table 1:** In vitro IC_50_ values of duocarmycin derivatives in different cell lines. N/A = not applicable.

Type of Cancer	Cell Line	Duocarmycin Analogs	Incubation Time	Assay/Endpoint	IC_50_	Reference
Human uterine cervix carcinoma	HeLa S_3_	DUMA	1 h	Growth Inhibition	0.12 nM	[49]
					0.0058 nM	[48]
					0.006 nM	[28]
		DUMB1	1 h	Growth Inhibition	0.035 nM	[28]
		DUMB2	1 h	Growth Inhibition	0.1 nM	[28]
		DUMC1	1 h	Growth Inhibition	8.5 nM	[28]
		DUMB2	1 h	Growth Inhibition	0.57 nM	[28]
		DU-86	1 h	Growth Inhibition	0.23 nM	[49]
					0.0052 nM	[48]
		DSA	1 h	Growth Inhibition	0.00069 nM	[48]
Human glioblastoma	U-138	DSA	14 days	Colony Formation	0.0018 nM	[5]
			72 h	Growth Inhibition	0.4 nM	[5]
	U-251	DUMC2	N/A	Cytotoxicity Profiles	0.794 nM	[39]
		DSA	N/A	Cytotoxicity Profiles	~0.63 nM	[39]
Human acute myeloid leukemia	Molm-14	DSA	72 h	MTT	0.01112 nM	[50]
			48 h	Annexin-V Staining	~0.1 nM	[50]
			7 days	Colony Formation	0.02 nM	[50]
			N/A	Cytotoxicity Profiles	~0.001 nM	[39]
		DUMC2	N/A	Cytotoxicity Profiles	~1.58 nM	[39]
	HL-60	DSA	72 h	MTT	0.1227 nM	[50]
			48 h	Annexin-V Staining	~0.25 nM	[50]
			7 days	Colony Formation	0.05 nM	[50]
			N/A	Cytotoxicity Profiles	~0.0158 nM	[39]
	Molt-4	DUMC2	N/A	Cytotoxicity Profiles	~0.001 nM	[39]
		DSA	N/A	Cytotoxicity Profiles	~0.001 nM	[39]
Mouse lymphocytic leukemia	L1210	DSA	N/A	Cytotoxicity Profiles	~0.001 nM	[39]
Human renal adenocarcinoma	786-0	DUMC2	N/A	Cytotoxicity Profiles	~63.1 nM	[39]
		DSA	N/A	Cytotoxicity Profiles	~0.001 nM	[39]
Human prostatic adenocarcinoma	PC-3	DUMC2	N/A	Cytotoxicity Profiles	~3.16 nM	[39]
		DSA	N/A	Cytotoxicity Profiles	~0.00126 nM	[39]
Human pancreatic adenocarcinoma	Capan-1	DUMC2	N/A	Cytotoxicity Profiles	~0.0631 nM	[39]
		DSA	N/A	Cytotoxicity Profiles	~0.001 nM	[39]
Human colorectal adenocarcinoma	HT-29	DUMC2	N/A	Cytotoxicity Profiles	~31.6 nM	[39]
		DSA	N/A	Cytotoxicity Profiles	~5 nM	[39]
Human breast adenocarcinoma	MCF-7	DUMC2	N/A	Cytotoxicity Profiles	~79.4 nM	[39]
		DSA	N/A	Cytotoxicity Profiles	~0.79 nM	[39]
Human ovarian adenocarcinoma	OVCAR-3	DUMC2	N/A	Cytotoxicity Profiles	~1 nM	[39]
		DSA	N/A	Cytotoxicity Profiles	~5 nM	[39]
Human lung adenocarcinoma	H322	DUMC2	N/A	Cytotoxicity Profiles	~3.98 nM	[39]
		DSA	N/A	Cytotoxicity Profiles	~2.51 nM	[39]
Human lung squamous cell carcinoma	UCLA-P3	DUMC2	N/A	Cytotoxicity Profiles	~25.1 nM	[39]
		DSA	N/A	Cytotoxicity Profiles	~2 nM	[39]
Human cervical squamous cell carcinoma	SiHA	DUMC2	N/A	Cytotoxicity Profiles	~31.6 nM	[39]
		DSA	N/A	Cytotoxicity Profiles	~0.4 nM	[39]
Human breast ductal carcinoma	BT-549	DUMC2	N/A	Cytotoxicity Profiles	~0.891 nM	[39]
		DSA	N/A	Cytotoxicity Profiles	~0.063 nM	[39]
Human melanoma	SK-MEL-28	DUMC2	N/A	Cytotoxicity Profiles	~7.94 nM	[39]
		DSA	N/A	Cytotoxicity Profiles	~1 nM	[39]
Human amelanotic melanoma	M24-MET	DUMC2	N/A	Cytotoxicity Profiles	~8.91 nM	[39]
		DSA	N/A	Cytotoxicity Profiles	~1.26 nM	[39]
Human mammary epithelial cells	HMEC	DUMC2	N/A	Cytotoxicity Profiles	~2.51 nM	[39]
		DSA	N/A	Cytotoxicity Profiles	~0.5 nM	[39]
Human dermal fibroblasts	NHDF	DUMC2	N/A	Cytotoxicity Profiles	~1000 nM	[39]
		DSA	N/A	Cytotoxicity Profiles	~0.001 nM	[39]
Human B lymphoblast	RPMI 7666	DUMC2	N/A	Cytotoxicity Profiles	~1000 nM	[39]
		DSA	N/A	Cytotoxicity Profiles	~316 nM	[39]
Hamster epithelial-like ovarian cells	CHO	DUMC2	N/A	Cytotoxicity Profiles	~50.1 nM	[39]
		DSA	N/A	Cytotoxicity profiles	~15.8 nM	[39]

**Table 2:** Progress of synthetic analogs of duocarmycin in dlinical trials.

Drug Name	Clinical Trial	Results	Reference
Adozelesin	Phase I	29 patients with refractory solid tumors or lymphomas participated. Administered via a 24 h continuous intravenous infusion, with initial treatments every 3 weeks, later extended to every 6 weeks due to prolonged myelosuppression, including thrombocytopenia and granulocytopenia. Dose range: 30 μg/m^2^ to 150 μg/m^2^. The maximum tolerated dose was 100 μg/m^2^. No antitumor responses were observed.	[83]
Adozelesin	Phase I	26 patients with refractory solid tumors participated. Patients received increasing doses of adozelesin through brief intravenous infusions every three weeks. The maximum tolerated dose was 188 mg/m^2^. Dose-limiting toxicity was myelosuppression, including thrombocytopenia and leukopenia. Nonhematologic toxicities were typically mild, with the most common adverse effects including fatigue (36%), local reactions at the infusion site (24%), nausea or vomiting (20%), and hypersensitivity reactions (16%). No antitumor responses were observed.	[84]
Adozelesin	Phase I	33 patients with refractory solid tumors who received prior cytotoxic treatment participated. Administered via 10 min IV infusion for 5 consecutive days every 3 weeks. Dose range: 6–30 mcg/m^2^/day. The maximum tolerated dose was 30 mcg/m^2^/day. Dose-limiting toxicity was myelosuppression, including thrombocytopenia and leukopenia. Significant toxicity was an anaphylactoid syndrome and occurred in 2 patients. A partial response was observed in a patient with refractory soft tissue sarcoma.	[85]
Adozelesin	Phase I	47 patients with solid malignancies participated. Adozelesin was administered via IV infusion every 6 weeks. Successive dose levels used were 10, 20, 33, 50, 70, 105, 120, 150, and 180 μg/m^2^. Dose-limiting toxicity was myelosuppression. The maximum tolerated dose was 180 μg/m^2^. A minor response was observed with a 4-month treatment in a patient with melanoma who had prior cytotoxic treatment.	[86]
Adozelesin	Phase II	Patients with untreated metastatic breast cancer participated. Out of 17 enrolled patients, 14 were evaluated. The trial aimed to enroll at least 25 patients but was stopped early due to slow enrollment and limited efficacy. Administered via IV infusion at a starting dose of 150 µg/m^2^ for 10 min, repeated every 4 weeks, for up to 1 year of treatment. The responses observed were one partial response (7%), three stable diseases (22%), and ten progressive diseases (71%). The most frequent adverse side effect was myelosuppression, and one patient died of pulmonary complications.	[87]
Carzelesin	Phase I	25 patients with various solid tumors participated. Administered via IV infusion for 10 min, daily for 5 consecutive days, repeated every 4 weeks. Starting dose was 12 µg/m^2^/day and escalated by 20–30% increments. The maximum tolerated dose was 40 µg/m^2^/day. Dose-limiting toxicities were neutropenia and thrombocytopenia. Pharmacokinetic findings: carzelesin was detectable up to 1 h post-infusion. U-76,073 detectable for up to 30 min at the maximum tolerated dose. U-76,074 was not detectable. Partial remission lasting 8 months observed in one patient with hepatocellular carcinoma at 40 µg/m^2^/day.	[88]
Carzelesin	Phase I	31 patients participated. Administered via IV infusion for 10 min. Doses were escalated from 24 to 48, 96, 130, 150, 170, 210, 250, and 300 μg/m^2^. Carzelesin elimination half-life: 23 ± 9 min (mean value ± SD). The intermediate metabolite U-76,073 was quickly formed, whereas the active metabolite U-76,074 was detected only at higher dose levels for an abbreviated period. The plasma levels of carzelesin, U-76,073, and U-76,074 were well above their respective IC_50_ values obtained in in vitro clonogenic assays. However, these levels were significantly lower than those observed in preclinical studies in mice. A substantial discrepancy in plasma clearance was observed between patients from two different institutions, suggesting potential issues with drug administration in one of the institutions. Myelosuppression was the main adverse side effect.	[89]
Carzelesin	Phase I	51 patients with advanced solid tumors participated. Tumor types included colorectal, melanoma, head and neck, sarcoma, renal, and others. Administered via IV infusion for 10 min, repeated every 4 weeks. Patients received a median of two administrations (range 1–5) at one of nine dose levels: 24, 48, 96, 130, 150, 170, 210, 250, and 300 μg/m^2^. The primary dose-limiting toxicities were neutropenia and thrombocytopenia. These toxicities were delayed in onset, prolonged, and cumulative. The active metabolite U-76,074 was detectable at higher dose levels but only for short periods. Out of the 51 patients, 34 had progressive disease, and 9 had stable disease as the best overall response.	[90]
Carzelesin	Phase II	140 patients participated. Administered as a first-line treatment for those with colorectal cancer, gastric cancer, and melanoma. Administered as a second- or third-line chemotherapy for patients with breast, ovarian, head and neck cancers, and non-Hodgkin’s lymphoma. Carzelesin was given via a bolus infusion at a dose of 150 μg/m^2^ every four weeks. Myelotoxicity was the most frequent adverse effect. Grades 3 and 4 leukopenia occurred in 18.6% of the treatment courses, neutropenia in 20.3%, thrombocytopenia in 16.2%, and anemia in 8.7%. Only one partial response was observed in a patient with melanoma. Carzelesin did not demonstrate efficacy in the types of tumors studied.	[91]
Bizelesin	Phase I	19 patients participated. Tumor types included colorectal, upper gastrointestinal, breast, and others. Doses ranged from 0.1 to 1 μg/m^2^. Administered via IV bolus injection. Dose-limiting toxicity was neutropenia. The maximum tolerated dose was 0.8 μg/m^2^ No objective responses were observed in the patients.	[92]
Bizelesin	Phase I	62 patients with advanced solid malignancies participated. Administered as a single IV bolus injection every 4 weeks. 185 courses at eight dose levels ranging from 0.1 to 1.5 µg/m^2^. The maximum tolerated dose was 0.8 μg/m^2^. The primary adverse effect was myelosuppression including neutropenia. No partial or complete antitumor responses were documented overall. A notable 40% reduction was observed in a patient with advanced ovarian carcinoma. The treatment was discontinued due to prolonged thrombocytopenia, but the antineoplastic response lasted for 8 months, making the total response duration 24 months.	[93]
Pibrozelesin (KW-2189)	Phase I	22 patients with solid tumors that were refractory to standard chemotherapy participated. Administered via IV bolus daily for 5 days every 6 weeks; patients received a total of 31 cycles. Leukopenia, neutropenia, and thrombocytopenia were the dose-limiting toxicities. The active metabolite DU-86 was not consistently found in patient plasma.	[94]
Pibrozelesin (KW-2189)	Phase II	40 patients with metastatic renal cell carcinoma participated. Patients were administered 0.4 mg/m^2^ via IV infusion for Cycle 1, with cycles repeated every 5 to 6 weeks and dose escalations to 0.5 mg/m^2^ if no significant toxicity or disease progression was observed. Hematological toxicity was dose-limiting. No objective response was observed. The median time to disease progression was 3.7 months, and the median survival time was 8.2 months.	[95]
Pibrozelesin (KW-2189)	Phase II	30 patients without prior cancer treatments and 15 patients with prior treatments participated. Patients were with metastatic melanoma. Administered at 0.5 mg/m^2^ to previously untreated patients and 0.4 mg/m^2^ to the previously treated. Treatment was administered intravenously on day 1 of a 6-week cycle. Neutropenia and thrombocytopenia were dose-limiting toxicities. The overall objective response rate was 17%. One previously treated patient was still alive 2.9 years after study entry. Three previously untreated patients were still alive 1.6, 2.3, and 2.9 years after study entry. The 1-year survival rate was 23% for untreated patients and 27% for previously treated patients.	[96]
Pibrozelesin (KW-2189)	Phase II	16 patients with metastatic hepatocellular carcinoma participated. Administered via IV bolus; 0.5 mg/m^2^ on day 1 of a 6-week cycle. Due to hematologic toxicity, the dose was reduced to 0.375 mg/m^2^ for 5 patients. The 11 patients in the high-dose group received a median of 3 treatment cycles, ranging from 1 to 9 cycles. Severe hematologic toxicities in this group included thrombocytopenia (82%), neutropenia (82%), leukopenia (64%), and anemia (36%). The 5 patients in the lower-dose group received a median of 1 treatment cycle, ranging from 1 to 4 cycles. Severe hematologic toxicities in this group included neutropenia (60%), leukopenia (40%), and thrombocytopenia (20%). The higher-dose group had a median progression-free survival of 20 weeks and a median survival time of 11.4 months. The lower-dose group had a median progression-free survival of 6.6 weeks and a median survival time of 9 months.	[97]
